# Novel Approach to Tooth Chemistry. Quantification of the Dental-Enamel Junction

**DOI:** 10.3390/ijms22116003

**Published:** 2021-06-02

**Authors:** Andrzej Kuczumow, Renata Chałas, Jakub Nowak, Janusz Lekki, Katarzyna Sarna-Boś, Wojciech Smułek, Maciej Jarzębski

**Affiliations:** 1 ComerLab Dorota Nowak, Radawiec Duży 196, 21-030 Motycz, Poland; andrzej.kuczumow@gmail.com (A.K.); kubit75@gmail.com (J.N.); 2 Department of Oral Medicine, Medical University of Lublin, 20-093 Lublin, Poland; renata.chalas@umlub.pl; 3 Henryk Niewodniczański Institute of Nuclear Physics, Polish Academy of Sciences, Department of Applied Spectroscopy, 31-342 Krakow, Poland; janusz.lekki@ifj.edu.pl; 4 Department of Dental Prosthetics, Medical University of Lublin, 20-093 Lublin, Poland; katarzyna.sarna-bos@umlub.pl; 5 Institute of Chemical Technology and Engineering, Poznan University of Technology, 60-965 Poznań, Poland; wojciech.smulek@put.poznan.pl; 6 Department of Physics and Biophysics, Poznań University of Life Sciences, 60-637 Poznań, Poland

**Keywords:** dental-enamel junction (DEJ), chemical microanalysis, mechanical properties, optical pattern, energy of apatite transformation

## Abstract

The dentin-enamel junction (DEJ) is known for its special role in teeth. Several techniques were applied for the investigation of the DEJ in human sound molar teeth. The electron (EPMA) and proton (PIXE) microprobes gave consistent indications about the variability of elemental concentrations on this boundary. The locally increased and oscillating concentrations of Mg and Na were observed in the junction, in the layer adhering to the enamel and covering roughly half of the DEJ width. The chemical results were compared with the optical profiles of the junction. Our chemical and optical results were next compared with the micromechanical results (hardness, elastic modulus, friction coefficient) available in the world literature. A strong correlation of both result sets was proven, which testifies to the self-affinity of the junction structures for different locations and even for different kinds of teeth and techniques applied for studies. Energetic changes in tooth strictly connected with crystallographic transformations were calculated, and the minimum energetic status was discovered for DEJ zone. Modeling of both walls of the DEJ from optical data was demonstrated. Comparing the DEJ in human teeth with the same structure found in dinosaur, shark, and alligator teeth evidences the universality of dentin enamel junction in animal world. The paper makes a contribution to better understanding the joining of the different hard tissues.

## 1. Introduction

Recently, an increasing number of studies have been focusing on the mechanical properties of the dentin-enamel junction (DEJ). While challenging, micro and nano levels of DEJ analyses provide valuable information. Intermolecular interactions at the nanometer level and interactions of nano- and microcrystalline structures strongly determine the properties of each material, also biological ones. The bone and the dental structures are special examples of nano- and micro-composite materials, of which the constituent particles and macromolecular structures are both of organic and inorganic nature [1,2].

The dentin-enamel junction has attracted the attention of scientists for years. Dentists and biomaterial science specialists have been interested in the determination of the structure and role of this small fragment of the tooth. The tooth is a partially living organ (role of pulp-dentin complex), strong and well-protected (role of enamel and cementum). In between, the narrow, clearly distinctive layer is located, strictly joining the abovementioned phases. Together with the function of integration of the phases in the tooth structure, this strip avoids expansion of mechanical pressures inside the dentin and prevents sliding of the phases each other. The DEJ layer provides the integrity to the agglomerates of both above phases. As it was explained by Arsenault and Robinson [3] and confirmed by Fang et al. [4], the matrix composed of organic matter plays a basic role in setting the continuity between dentin and enamel crystallites along the DEJ. The dentin and enamel are composed of roughly the same inorganic matter (modified hydroxyapatites), supplemented with an organic matrix, the latter different in quality and quantity in both phases. The need for research on the DEJ and its considerable role results from the awareness that two dissimilar phases should be joined in a durable way to buffer the spread of cracks from the damaged enamel into the dentin zone [5,6]. At the same time, the possibility of material transport between dentin and enamel through the junction should be guaranteed. The optical images and the sizes of the DEJs are surprisingly uniform across various species from different locations and epochs such as extinct dinosaurs, horses, and recent beaver [7,8,9], with exception of the inverted coloration of dentin and enamel in living tissues and fossils [10]. However, the estimation of the size of the junction in humans varied greatly depending on the kind of tooth as well as the method of observation and measurement, with the data ranging from 2–3 µm up to several tens of µm [11,12,13]. For example, it was found in the study by Gallagher et al. [14] that the DEJ width derived from their micromechanical measurements was in the range 4.7–6.9 µm, while it was 7.6–8.5 µm as estimated from micro-Raman measurements. Another important factor of the variability in DEJ size is the scalloped structure of the tooth which makes the width vary in different places. However, our thesis is that the linear cross-sections through DEJ show self-affinity, i.e., they look extremely similar, independent of the spectral and spatial resolution of the instrument used for the studies. As a rule, a single method of measurement was considered for the estimation of the width. A complex topographical structure was observed at the junction, with some convex structures directed inside the dentin and some cavities in the enamel on the junction-enamel boundary [13,15]. The spread of the parallel organic fibrils of collagen I can be detected via SEM observations [16]. The fibrils expand from the dentin towards the dentin-enamel junction where they enter at a shallow depth within the enamel zone. The problem in studies on DEJ is that the layer cannot be isolated from the enamel and the dentin.

The progress in atomic force microscopy (AFM) associated with nano-indentors has enabled measurements of the microhardness and elastic modulus [17,18,19,20], while an attachment of the nanoscratch tester provided the opportunity to measure the friction coefficient on the border of the DEJ-dentin [21]. There have been attempts to maps the results of nanoindentation [22,23], regardless of the challenges due to the small width of the DEJ. It is worth considering that AFM, although a typical nanoscale device, was applied at a microscale level in the measurements for microhardness and elastic modulus, except in the investigations described by Habelitz et al. [20]. It was due to the fact that the mechanical tests had damaged the material structure in the measured area on a micrometer range. Each next undisturbed measurement was only possible in a location some micrometers far away from the previous trial. The application of scanning acoustic microscopy (SAM) seems to be a non-damaging alternative for the mentioned mechanical tests [24]; however, it depends on spatial resolution of the method which is important for studies of DEJ.

Such an approach inclines towards linking the micromechanical tests with typical microchemical analyses and with the microoptical image. In the papers mentioned, the width between the extreme slope locations for a particular variable was considered as a rule as the equivalent to the width of the dentin-enamel junction. Another approach was demonstrated only in the paper by Schulze et al. [25]. Here, we attempt to demonstrate that one should take into account at least two mechanical features to delimit the DEJ. The mentioned mechanical features are linked to two different locations (walls) in the DEJ. Moreover, the mechanical parameters can be strictly coupled with the changes in the chemical composition, and two of such chemical changes characterize the DEJ. The chemical studies on the DEJ are made in this contribution using the Electron Probe Microanalyzer (EPMA), Proton Induced X-Ray Emission (PIXE), and Raman microprobe; the latter also analyzes the organic components [26]. In our previous articles [27,28], we presented the great effectiveness of microprobes in the detection of the elements on a microscale for other kinds of biological microstructures. There are other interesting publications about the possibility of spectral techniques in studies on biomaterials [29,30]. The same effectiveness of those methods should be observed for the DEJ zone. The shape and size of the DEJ can be reconstructed in an optical image.

The microchemical (proton and Raman microprobes) studies on the DEJ, although significant, were as a rule made hitherto without links to the micromechanical studies [31,32,33]. Computed tomography was only recently applied to reconstruct 3D structure of bovine tooth DEJ [34]. Little was known about the chemical components inside the zone. This contribution also aims to supplement our knowledge in the field. The chemical changes on the boundaries must have an influence on the optical and mechanical features of the material.

Our initial suppositions about expected changes in DEJ zone are as follows: density, Ca and P contents, hardness, elastic modulus decrease from enamel to dentin side due to greater contents of apatite in the enamel, and organic matter and friction—in opposite side, due to greater amount of organic matter and greater inhomogeneity (due to tubule structure) on dentin side.

Thus, the main objective of this study is to characterize the DEJ, taking into account our own microchemical, optical, and energetical results. The next aim is correlating our data with micromechanical data available in the world literature. Coupling of those datasets is significant also, as well as establishing the correlations between each other set.

The distribution of material is as follows: presentation of our own microchemical and optical data—addition of available micromechanical data—our estimation of energetic features of DEJ.

## 2. Results and Discussion

### 2.1. Cross-Sectional Outline of the DEJ

**Figure 1:**
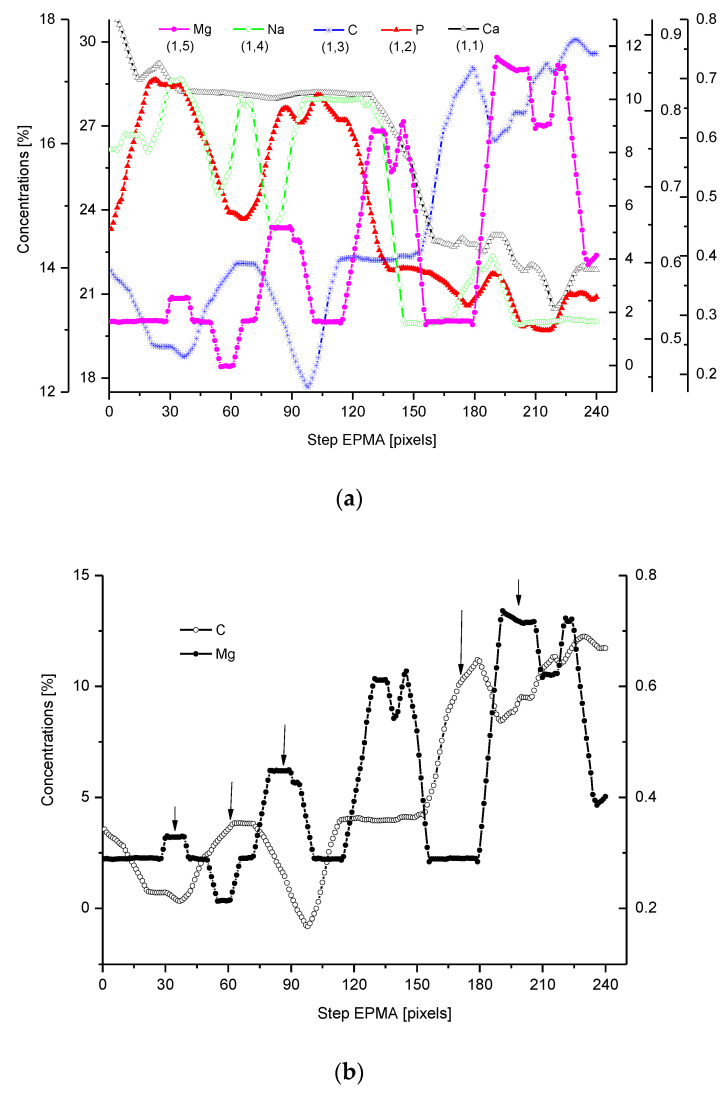

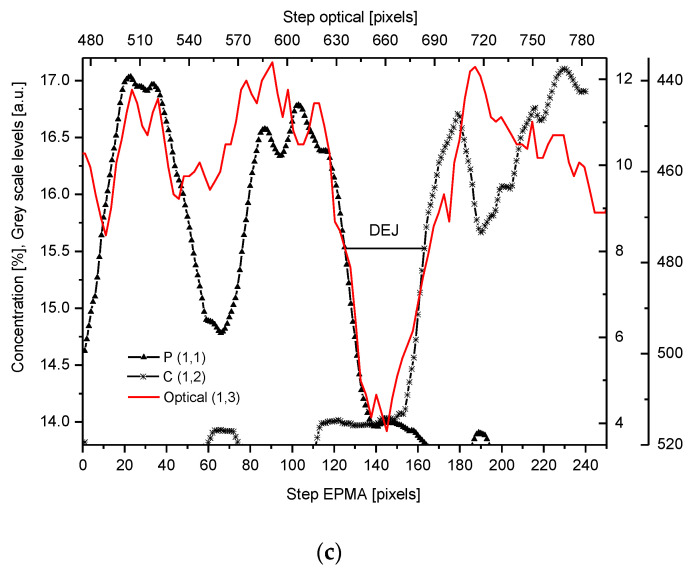
(**a**) Results of EPMA measurements along the DEJ zone. The direction is from the enamel (left) towards the dentin and always the same direction is preserved in the next figures (where relevant), and the length is equal to 250 µm. 1 step = 1 µm. (**b**) Sequence of C and Mg variability, in majority cases inversely directed. (**c**) Outline of the DEJ space with optical signal and in parallel by two spectral signals of P and C. On multiscale figures the numerical markers were introduced where the first number denotes x-axes, calculated in the system down-up and the second one y-axes, calculated from left to right side of the figure.

**Figure 2:**
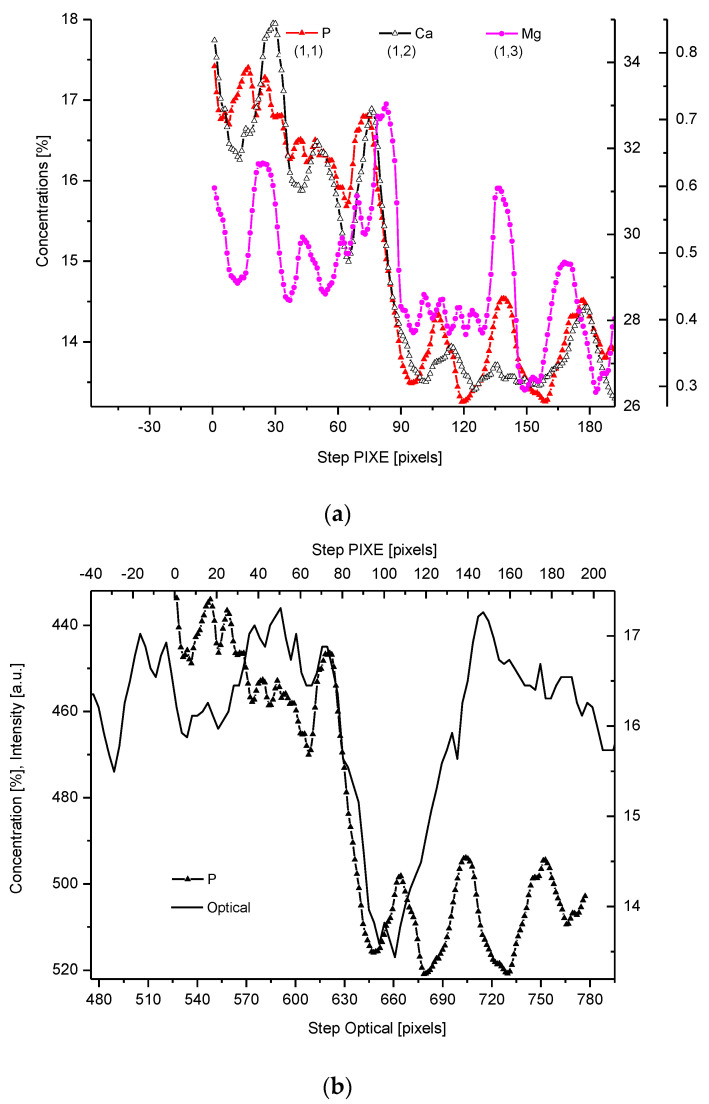

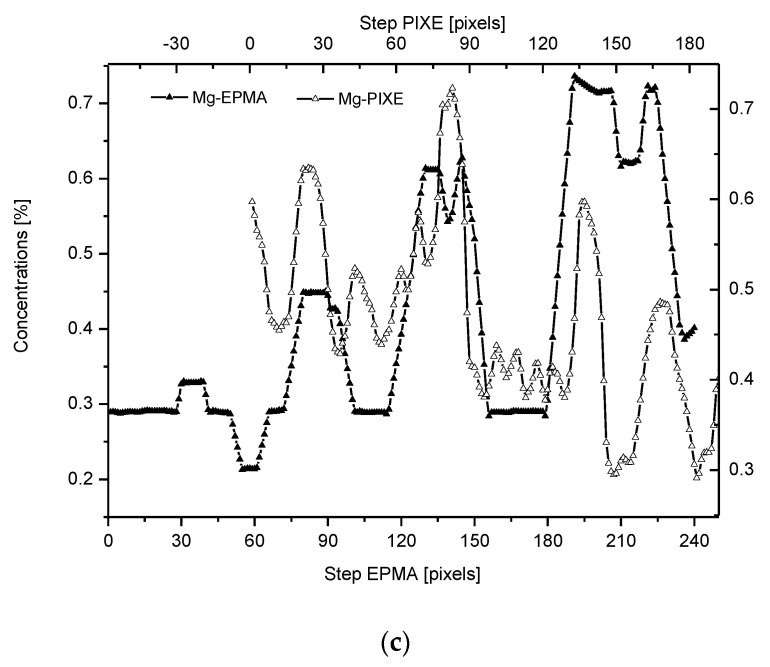
(**a**) Results of µ-PIXE measurements. 1 step = 1.25 µm. (**b**) Outline of the DEJ space with optical signal and with P signal, for left boundary only; please observe missing boundary DEJ-dentin due to the air path of measurements and inability of detection of carbon. (**c**) Parallelism of Mg measurements with the use of EPMA and µ-PIXE.

**Figure 3:**
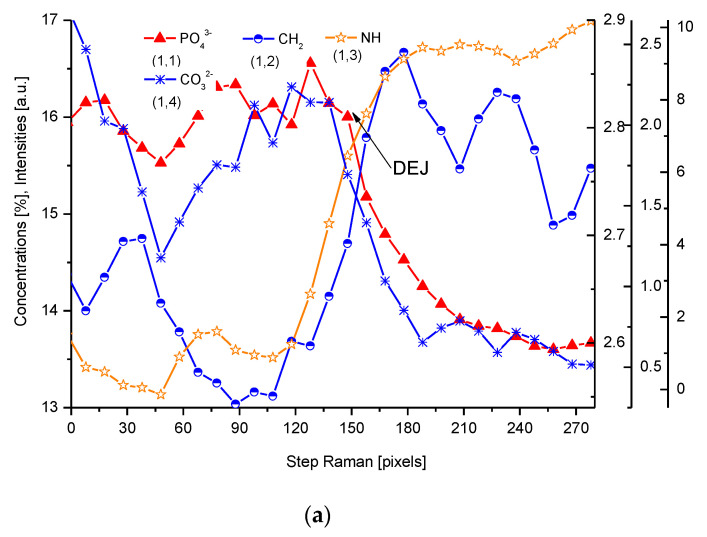

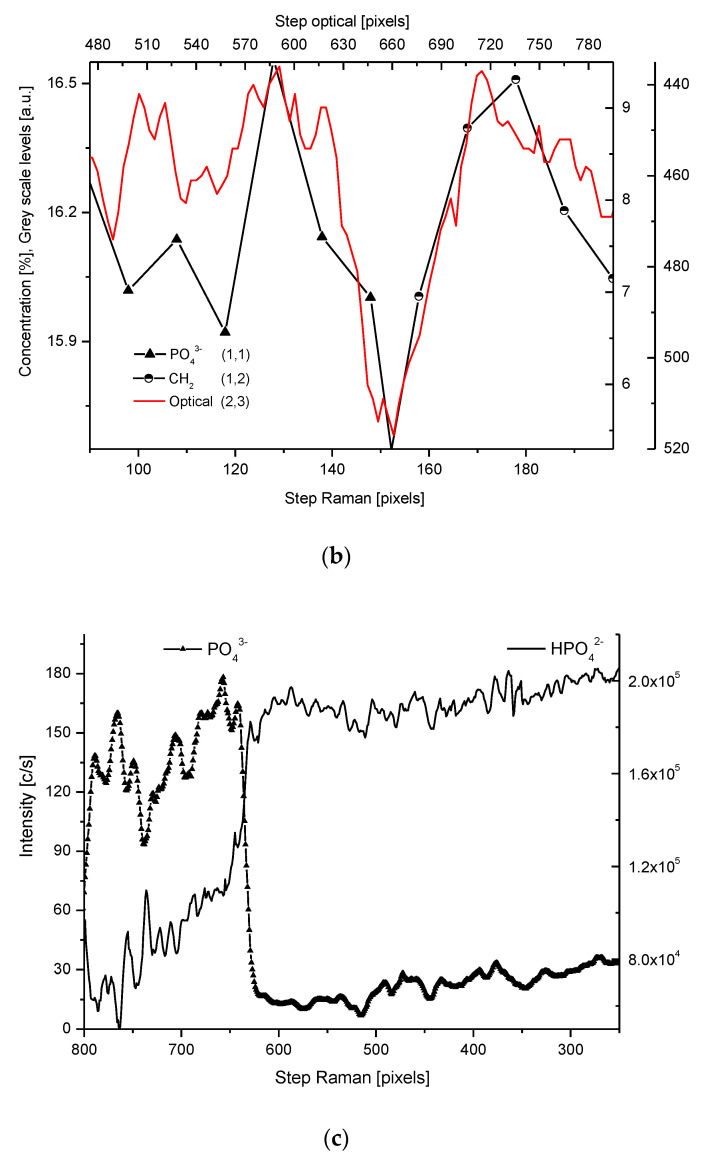
(**a**) Results of µ-Raman measurements around the DEJ. PO_4_^3−^-ν_1_ oscillation; CH_2_-ν symmetric oscillation; NH line; CO_3_^2−^, sub. B; 1 step = 1.61 µm; (**b**) Outline of DEJ space with optical and Raman PO_4_^3−^ and CH_2_ signals. (**c**) Divergent spatial profiles of PO_4_^3−^ and HPO_4_^2−^ ions, with visible DEJ.

We have collected three sets of results from the electron probe microanalysis (EPMA), µ-particle induced X-Ray emission (PIXE), and µ-Raman measurements. The results are presented in Figure 1, Figure 2 and Figure 3, respectively. All the scans were carried out at distances up to 250 µm, centered around the DEJ, to cover fully the DEJ zone together with surrounding regions of enamel on one side and dentin on the other side. Scanning for P, Ca, C, Na, and Mg was possible with EPMA, using the Kα1 lines of the elements and for carbon-Kα. Here, in Figure 1a, we could observe the clear anticorrelation between the concentrations of P and C in the enamel layer, the DEJ zone, and the first layer of the dentin, which testifies to mainly the inorganic, carbonate form of the C in this zone. At the same time, the levels of Ca concentrations seemed to be stable. Where the carbonate ion was included in a greater amount into the apatite structure, an automatic deficit of the phosphate ions occurred [35], and it could testify to the “B-type” substitution of CO_3_^2−^ ion in the previous PO_4_^3−^ site [24]. One can suppose that it occurred mainly in a direction oblique to the “c” axis of the apatite crystal, i.e., on a × a plane. This relationship is especially pronounced in the first layer of the enamel, adhering to the DEJ. The mentioned relationship is much more obscure in the deeper dentin locations, due to the mainly organic form of the carbon here. One can observe very clear oscillations in the composition of magnesium along the whole scan length, with consecutive maxima distant by more or less 50 µm. In the dentin phase, Mg seems to be anticorrelated with the C signal, except the DEJ region, and it is shown in Figure 1b. Similarly, Na content also shows oscillations, although in locations other than those of Mg. Figure 1c shows the delimitation of the DEJ from these measurements, emphasized by the same delimitation derived from the optical microscope. There is an occasion to define the size of DEJ. Here, we estimate that such delimitation should be set between the halves of slopes on the enamel/DEJ and DEJ/dentin boundaries. Essentially, although not always, the half of the slope is equivalent to the inflexion points on the relevant curves (e.g., on the linear scan of Ca concentrations). It can be observed in Figure 1c, where crossbeam shows the width of DEJ touches on both ends the deflection points on optical, P and C curves.

The PIXE scans of P, Ca, and Mg were made (Figure 2). One should notice the very clear variable periodic oscillations in the P and especially Mg signals, the latter distant from one another by about 50 µm. Once more, the clear local maximum could be observed in the first layer of enamel, adhering to the DEJ. We can note some discrepancies between P and Ca signals, reaching 15 and 45 µm on the length axis. They can be attributed to the elevated levels of CO_3_^2−^ ions, not detected here due to the X-Ray detection threshold of the Si (Li) detector window. The profile of phosphor was superimposed on the optical profile (Figure 2b). This time, only the left side of the DEJ was represented among chemical signals, since the C signal was not observed. Both in the EPMA and PIXE measurements, the Mg elevated concentrations were located even beyond the slope of Ca concentration towards the DEJ, thus, in half of the DEJ zone (Figure 2c). If one compares the elemental scans from different methods, the correlation between the signals of the same element measured often fails a little. It is not unexpected since the signals were collected along the lines shifted by micrometers; they were collected from different depths (PIXE allows collecting signals from depths which are an order of magnitude greater than in EPMA). However, the drop/growth of the measured values on the DEJ-enamel and DEJ-dentin boundaries is a very stable factor in this environment.

For µ-Raman, the lines of PO_4_^3−^ (960 cm^−1^), C-H (~2935 cm^−1^), CO_3_^2−^, substitution B (1086 cm^−1^), and NH (3470 cm^−1^) were detected. The two first of abovementioned lines, necessary for delimitation of the DEJ, were compared with the optical profile (Figure 3). Interestingly, profiles of PO_4_^3−^ and HPO_4_^2−^ lines are completely opposite (Figure 3c). Hence, we might delimit the DEJ using those two lines.

The collection of chemical results by the use of three different methods is not a question of trivial repetition. The EPMA and PIXE measurements inform us about the inorganic constituents of the sample while µ-Raman gives some information about the inorganic (phosphates, carbonates) and the organic (CH_2_, NH_2_) components. The PIXE data are collected from deeper layers (useful signal range spans from the surface up to ~30 µm) than the data from EPMA (~3 µm) and are not so disturbed by the surface roughness. In addition, a greater number of trace and/or heavier elements can be detected more easily. Comparing the repeatability of the results of analyses made by different methods was not the least important aim.

The quality of the data was highest for µ-PIXE measurements, but due to the X-Ray detection threshold of the detector, the measuring of C and thus the delimitation of the DEJ-dentin boundary was impossible. Different data were used to supplement one another. None of the results were repeated in strictly the same location on a particular sample, due to different technical and preparative difficulties. The lateral deviations of scans were estimated as <3 µm. Thus, the scans express the self-affinity rather than the very rigorous identity of results.

**Figure 4:**
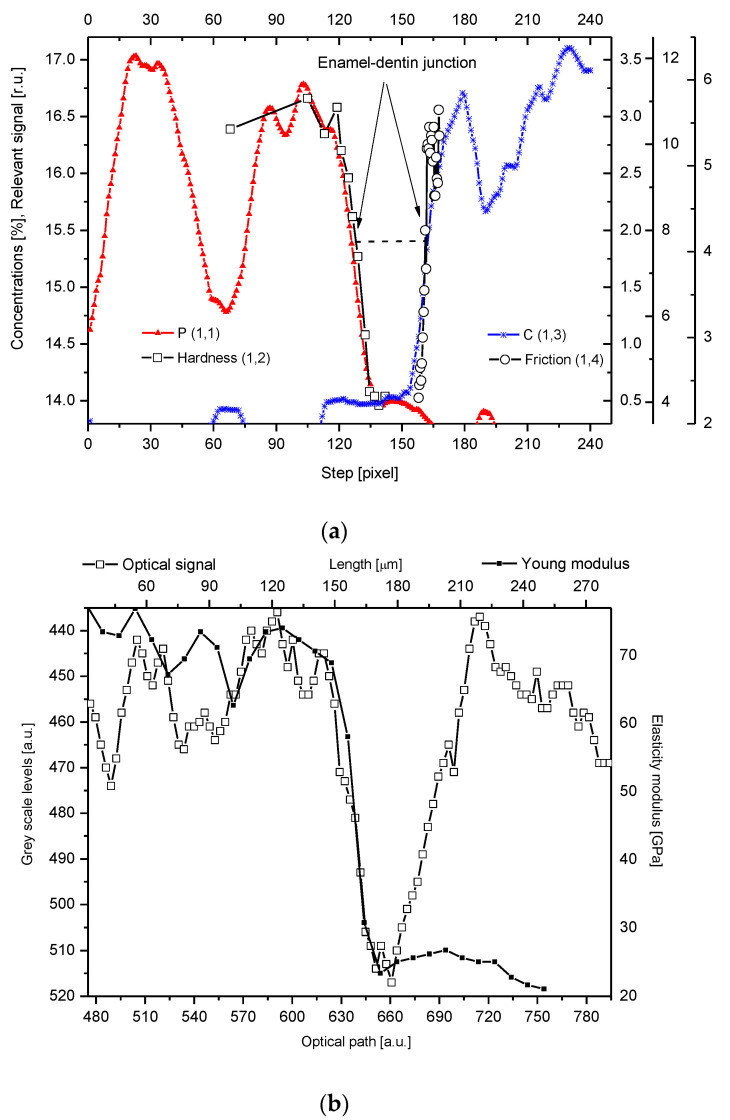

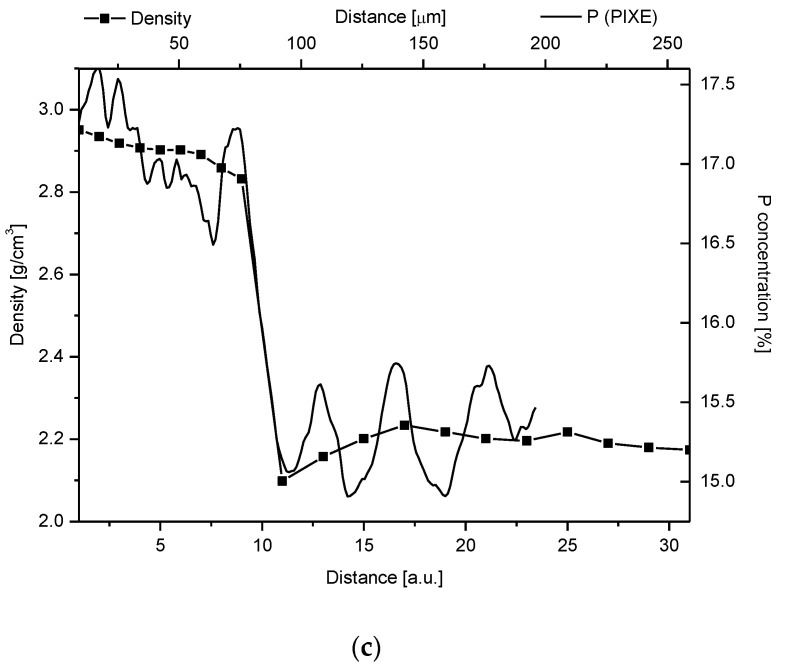
(**a**) Parallel delimitation of the DEJ zone with the EPMA measurements and micromechanical results. The micro-hardness and micro-friction results are adopted from Marshall et al. [12]. The horizontal dashed line delimits the width of the DEJ. (**b**) Superposition of Young modulus on the optical outline of DEJ. (**c**) Superposition of changes in density from Weatherell [35] on P measurement from PIXE.

One of the results is the observation of clear coincidence between the optical, microchemical, and micromechanical images of the DEJ. The DEJ differs clearly in its optical characteristics both from the enamel and the dentin (e.g., Figure 4b). It is undoubtedly promising, since the optical observation is incomparably easier than by other applied methods and is easy to use in diagnostics.

Some tendency can be observed for the widening of the DEJ in the images from the optical microscope, but it is probably associated with the poor level of the beam collimation in such a device. The beam collimation is the ultimate limit for the accuracy in the DEJ delimitation. We tried to compare our chemical and optical results with the available results of the ultrasonographic studies on the DEJ [36,37]. Some quite encouraging convergence of the general shapes of the DEJ was observed. On the other hand, the significant widening of the zone in the resonant ultrasound approach is discouraging. We understand that it results from the poor collimation and scattering of the ultrasound beam.

### 2.2. Discussion of DEJ Model

Our studies clarified some of the controversies concerning the DEJ. We suggested the following model of the junction, taking the results of our investigations into account and comparing them with the micromechanical results by Marshall et al. [12], Fong et al. [11], and finally, with our optical results. It is a zone with a width in the range of 15–35 µm (see Figure 1, Figure 2 and Figure 3, the split between P and C slopes in the halves of heights). Our results, although scattered for different samples (here not shown), were kept in the mentioned range. In our opinion, although it is a scatter in the estimation of the width, there is no support to the much lower widths suggested by Schultze et al. [25] and Balooch et al. [23]. Moreover, the higher values often result from the too great measurement steps (insufficient spatial resolution), as in an otherwise very interesting paper by Kolmas et al. [38]. The zone is of a complex structure. One can imagine both the existence of the enamel-DEJ boundary (it can be called the frontal wall) and a second boundary DEJ–dentin (the rear wall) and the middle zone—in-between. Therefore, not only the DEJ is different from the bulk enamel and dentin, but the adhering layers of enamel and dentin have their specific features as well. The DEJ imagination is shown in Figure 4. This figure is supported by our chemical studies from EPMA and the results of Marshall et al. [12] (hardness and friction)—tailored to our data. Please note that even with such mixed data (the teeth were taken from different European and American populations; ours are for molars, other for incisors) the variables can be matched very easily to one another. It acts as the self-affinity rule. From the enamel side, a rapid drop in the hardness occurs. This drop certainly coincides with the sudden decrease in the P and Ca (not shown here) concentrations. The drop in the Ca concentration is about 4% and in P about 2% over the distance of ~15 µm and corresponds to a drop in hardness by ~2.75 GPa over the same distance of 15 µm, and in a reduction of the elasticity modulus by ~50 GPa, as shown in Figure 4b (see Marshall et al. [12], their Figure 7). The density data, taken from Weatherell [35] and Anderson et al. [39] can also be tailored to our results which it is shown in Figure 4c.

**Figure 5:**
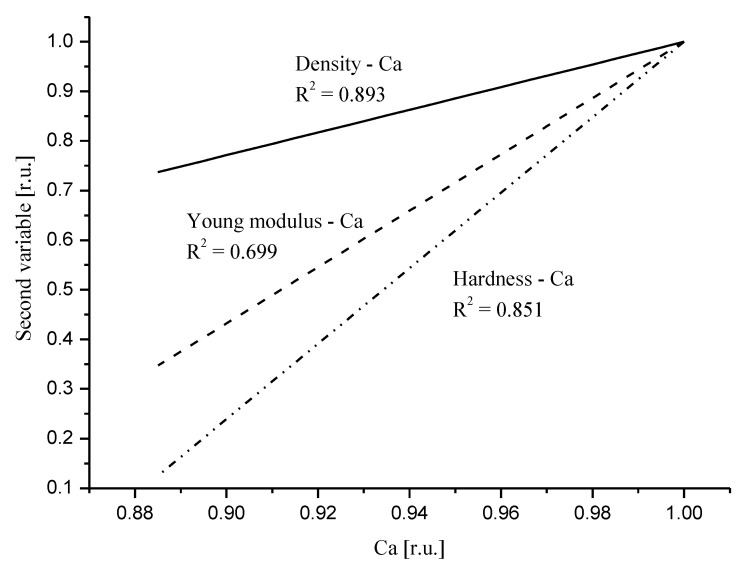
Comparison of the linear fits to the correlation relationships between relative values of density and Ca (solid line); Young modulus and Ca (dotted line); hardness and Ca (dot-drop line). Relevant equations are: RDen = −1.27 + 2.287 × [RCa]; RYM = −4.68 + 5.678 × [RCa]; RH = −6.61 + 7.609 × [RCa].

Here, we invoke Figure 3 from our previous paper [40]. We presented the relative influence of Ca drop on the mechanical parameters. Figure 5 in recent contribution resembles to much extent Figure 3 in the mentioned paper. The order of devastation occurring when the relative content of Ca diminishes is the same: density < Young modulus < hardness, but the degree of devastation is much greater for the DEJ. One must remember that the point of convergence of curves for relative values, which is equal to one, is another for the DEJ (it is point in enamel very close to DEJ) and another for enamel (it is point very close to the enamel–air boundary). Nevertheless, the influence of Ca drop is much more serious in the enamel–DEJ boundary than inside whole enamel and it occurs on a much smaller distance.

### 2.3. Energetic Relationships

**Figure 6:**
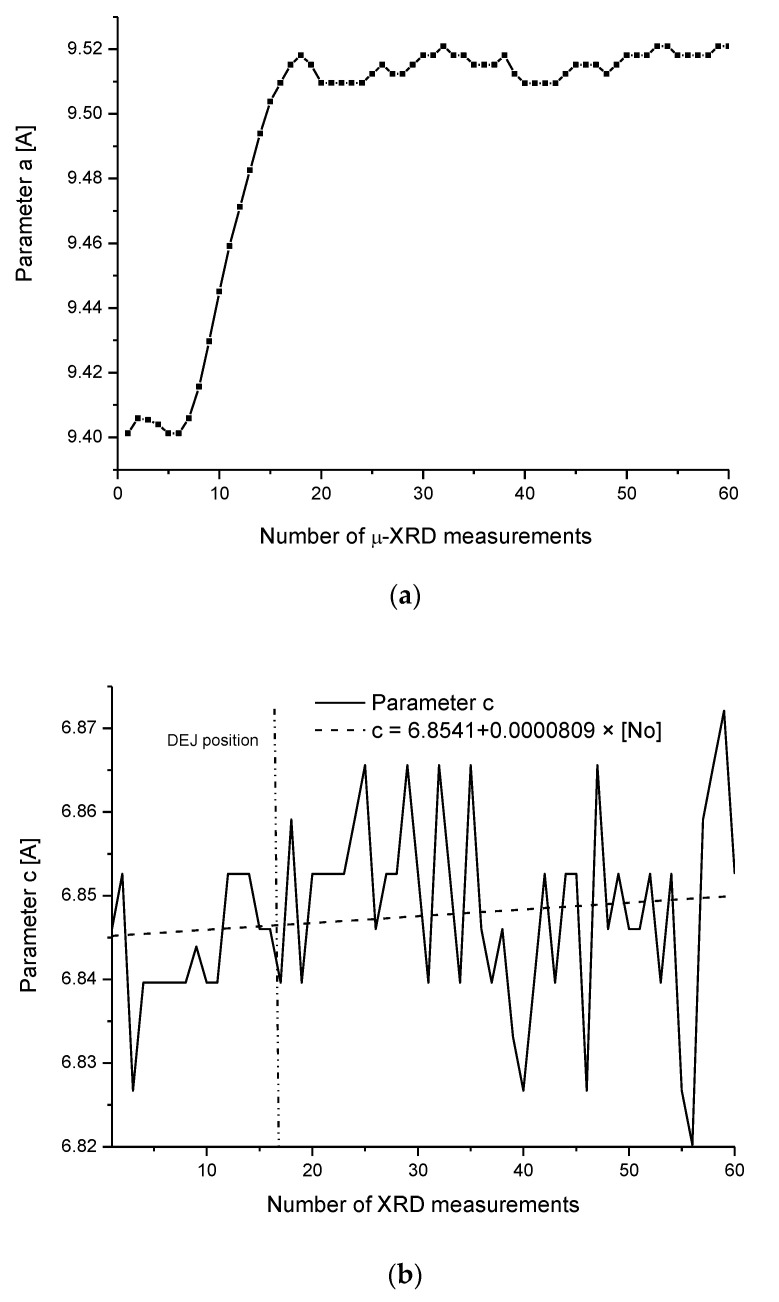

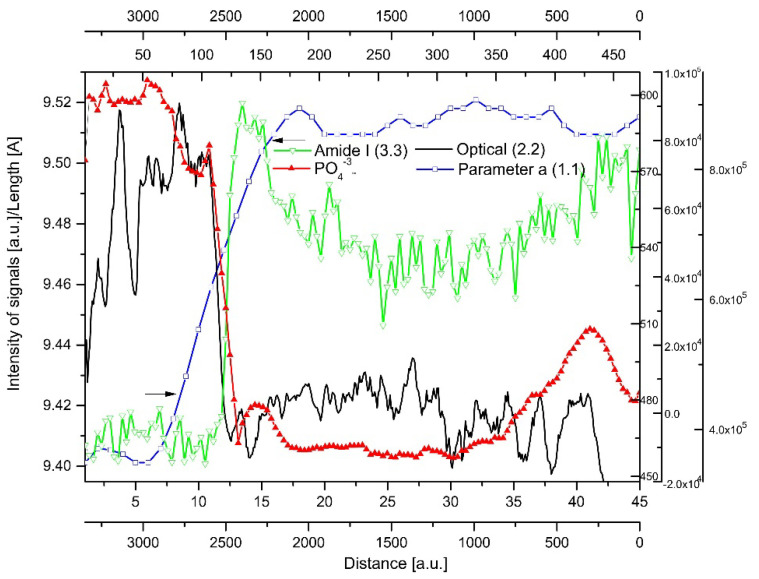

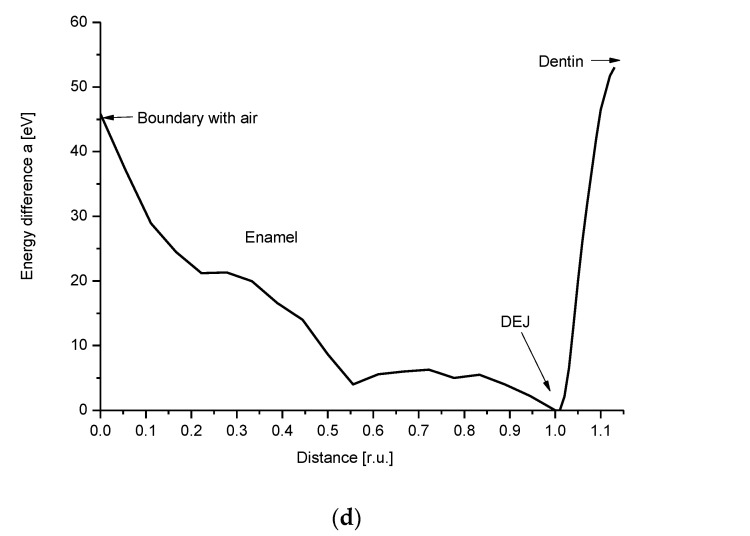
(**a**) Variability of crystallographic “a” parameter inside the tooth. (**b**) Generally faint variability of “c” parameter, even bearing in mind individual jumps. (**c**) Superposition of variability of parameter “a” on optical and chemical outline of the DEJ. Arrows show how the profile of “a” would be narrowed in the case of a better spatial resolution of the XRD system. (**d**) Energetic profile of whole enamel, DEJ, and fragment of dentin.

By using the procedure and equations we previously described from this series [40,41] for parameter “a”,

ΔE = 21.477/a_1_(1/sinΘ_1_ − sinΘ_2_)

or in equivalent version,

ΔE = 21.477/ sinΘ_1_(1/a_1_ − 1/a_2_)

we can calculate the energy difference which is necessary for the transformation of apatite crystal particle from dentin to that present in the DEJ. It is equal to 58.2 eV for changes along the “a” crystallographic axis if we take into account our own studies, 46.3 eV if one calculates according to the data by Al-Jawad et al. [42], and 54.9 eV as calculated due to the study of Hanlie et al. [43]. Figure 6d shows the imaging of how the whole enamel, plus DEJ zone, plus very first line of dentin looks if one observes the energetic variability expressed by transformation of “a” parameter. The variability along “c” axis is hardly visible (see Figure 6b). Additional transformation of the same particle to become the particle of apatite from first layer of enamel adhering to the DEJ demands only 4 eV along the “c” axis. The energetic well is strongly asymmetric; somebody crosses the DEJ. This difference in energy occurs along the dentin–DEJ boundary. Otherwise, this energetic barrier is large and equals to 9600 kJ/mol of apatite. Since this energy is negative on passing from dentin to the DEJ and since in enamel we have some minor increase in energy of transformation in direction of boundary with air, the apatites in DEJ zone are characterized by minimal energy. It stabilizes the DEJ zone and assures that the enamel apatite variability is possible only in enamel–air and blocked in DEJ–dentin direction. The changes of parameter “c” are not presented since they are negligible.

### 2.4. Correlations

**Figure 7:**
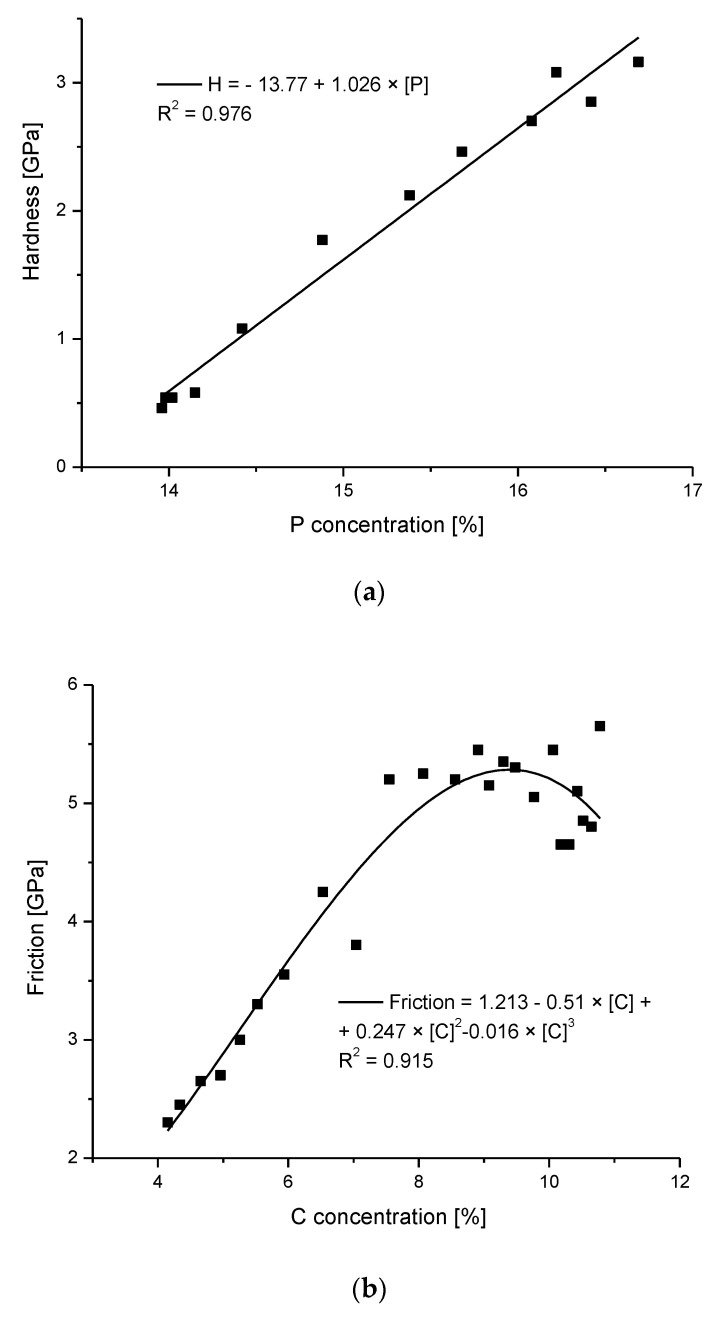

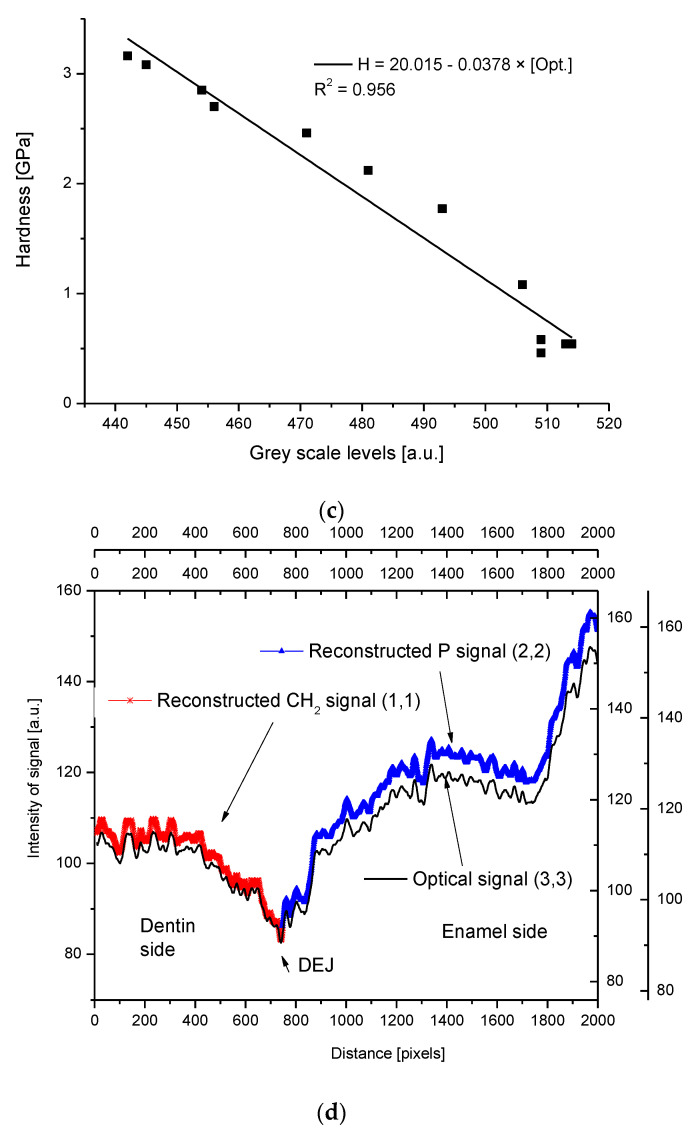
(**a**) Correlation between the drop in the phosphor concentration (EPMA) and the hardness, with the perfect linear fit to the points (P concentration from our studies, hardness adopted from Marshall et al., [12], as tailored in Figure 4). (**b**) Correlation between the increase in the CH_2_ concentration from Raman measurements and the friction coefficient (data from Marshall et al. [12]), recalculated and combined by us). (**c**) Correlation between hardness and optical signal. (**d**) Reconstruction of the DEJ from chemical signals transformed into optical signals using the relevant correlations and its comparison with the real optical signal.

Chemical and mechanical changes are perfectly correlated, and the correlation between our data on P and the data of Marshall et al. [12] on hardness is shown in Figure 7a. The above-mentioned hardness growth by 2.75 GPa results from the substitution of the organic matter in the dentin by much harder apatites in enamel. In addition, the inorganic material is now closely packed in the enamel.

From the dentin side, the increase in the C-H concentration (Raman measurements) is detected, and it is associated with an increase in the friction coefficient. Here, the slope of the micromechanical curve is even more rapid, with a very rapid increase of the friction coefficient on the dentin side (by 0.15 over a distance ~3–5 µm) and a significant, 6-fold increase in the C concentration according to studies of Marshall et al. [44] (see Figure 11 in that position) and a similar growth according to our studies (Figure 3). In this case, one should rather take into account a perfect correlation gained by Marshall et al. [12] since their measurements were made on the same sample (Figure 7b—our recalculation and presentation of joined results). Coming back to Figure 4, please note the clear image of the DEJ as gained from the comparison of EPMA chemical data and hardness and friction measurements. Here, the imaging of the DEJ by the combination of the chemical and mechanical data is presented. It can explain the drastic controversies between the authors concerning the width of the DEJ zone. It is clear from our presentation of the DEJ, that an individual who measures only the friction (or C) variations cannot treat the results as concerning the DEJ as such. He measures only the width of the DEJ-dentin wall; similarly, the measurement of the microhardness (either Ca or P) drop brings the dimension of the enamel-DEJ wall.

We compared the optical scans with the chemical results from EPMA (Figure 1b, Figure 2b and Figure 3b). The results are impressive, especially if one bears in mind that the measurements were made separately, at locations slightly different than those studied by EPMA. The optical limits can be compared with the PIXE results, but this time, only one boundary with the enamel can be revealed by the P signal. The DEJ can thus be delimited by the optical results (it is the fourth way of delimitation). Therefore, we infer that there must be a correlation not only between the optical [10] and chemical results but also between optical and mechanical results. Indeed, we found a correlation between the hardness and the change in the greyscale levels of the optical signal (Figure 7c). The correlation is presented in an independent and indirect way by Balooch et al. [23] (see Figure 3 in this position).

This interplay of chemical, mechanical, and optical data can serve as a source of interesting simulations. One can derive either the chemical or mechanical imagination of the DEJ from the optical profile of the DEJ or vice versa. Results of such a simulation are presented in Figure 7d, where signals of Ca and P are reconstructed from the previously calibrated optical profile.

### 2.5. Internal Structure of the DEJ and Adhering Zones

**Figure 8:**
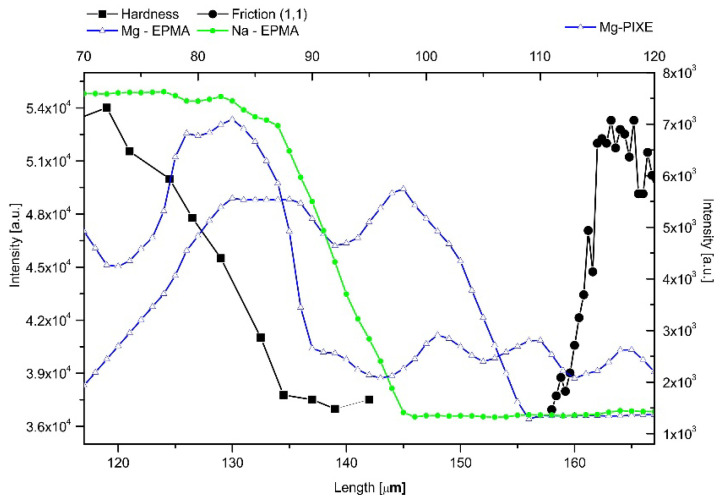

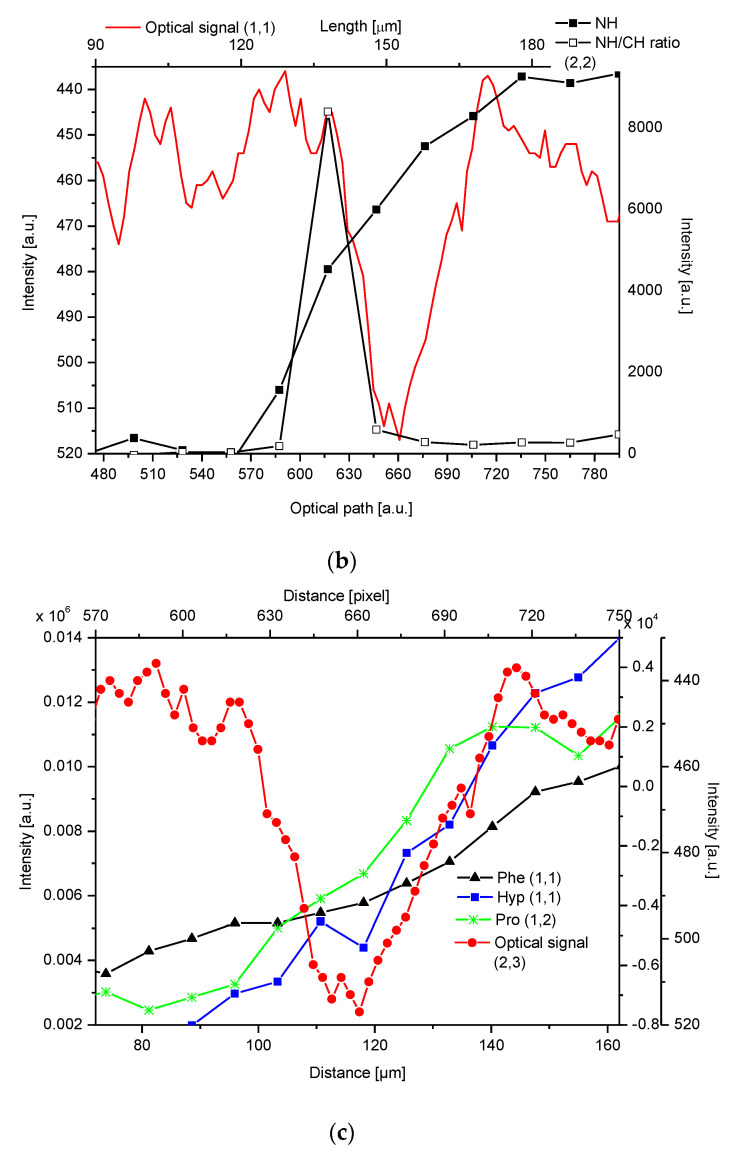

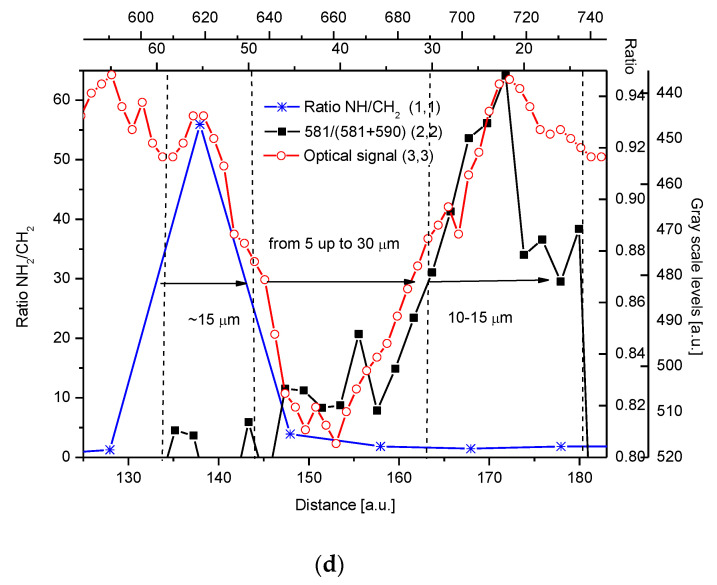
(**a**) The internal structure of the DEJ. Hardness and friction lines delimit here the DEJ zone. Mg is from EPMA and PIXE measurements and Na from EPMA measurements. Measurements were not made along the same line, but general tendencies are common. Values on y-axes were uniformized for better observation. (**b**) Superposition of the NH signal profile and the ratio of NH/CH_2_ profiles on the optical outline of the DEJ and Raman measurements. (**c**) Profiles of single amino acids present in collagen bundles within DEJ. (**d**) Variabilities of ratios of NH/CH_2_ and 581/(581 + 590) Raman lines mirroring left and right zones adhering to the DEJ.

The middle zone of the DEJ is not uniform either. One can observe the clearly elevated levels of Mg and Na in the part of the DEJ adhering to enamel (Figure 8a). Except for the EPMA measurements, where sometimes the zone covered by Mg is wider, the adhesive zone contains elevated levels of Mg and Na in the range of 10–12 µm, in approximation the half of the DEJ zone. In such an approach, one should recognize the role of Mg and Na in the reconstruction of the apatite crystals [45,46] and in potential stabilization of other phases [40], from compact crystals embedded in the organic matrix to the longitudinal rods.

The DEJ can also be delimited based on µ-Raman measurements, with the optical data superimposed. We paid attention to the fact that the DEJ zone presents itself as a zig-zagged strip if observed by the naked eye or in the optical microscope on the longitudinal cross-section of the tooth. If one extracts a linear scan from the image along any line transversal to the DEJ, a clear zone can be observed. It is shown in Figure 3b. Please note the strong and in some locations very good correlation between our chemical and optical results. Here, the optical results are comparable to the spatial resolution of the optical image (optical spatial resolution corresponds to 0.78 µm and 1 Raman point to 1.61 µm in our conditions). One can observe the general parallelism of the Raman and optical results. It demonstrates the fact that we can observe the changes in the DEJ and surrounding phases precisely with the optical microscope, unfortunately without any direct chemical information involved. Our results allow indirect passing from the chemical results to the optical observation. All this testifies to the general character of the two interfaces of the DEJ, that with enamel and that with the dentin.

Raman measurement also involves a new source of information about the internal structure of the DEJ. We can see that the value of the NH Raman signal drops much more mildly towards the boundary with the enamel than the value of the CH signal (compare Figure 3a and Figure 8b). It suggests that collagen fibrils from the bundle penetrating the DEJ on the dentin side and finishing flatly inside enamel are enriched in amino groups. The discovery is supported by a surprising jump in the value of NH/CH_2_ ratio in the narrow layer of the enamel adhering to the DEJ zone. Figure 8b,d shows that the ratio of NH/CH_2_ changes radically in the first layer of the enamel, adhering to DEJ, which is ~15 µm wide (Figure 8b). This enamel layer is probably the layer in which the fibrils have their ends, and then, there is significant surplus of NH groups, which possibly explains the overrepresentation of amino acids with two amino groups. It is confirmed by data from Figure 8c, where one can observe drops in signals of phenylalanine, hydroxyproline, and proline. Those amino acids can be easily detected with Raman spectroscopy. The profiles are similar to one another, but by no means identical, and it confirms that the proportion of amino acids changes along the DEJ. However, new studies by McGuire et al. [47] inform that collagen IV is present in the structure under interest, while collagen VII in the enamel layer adhering DEJ [48].

The ratio of oscillation intensities inside the bands belonging to mode ν_4_ for the phosphate group presents an interesting situation, due to E2 (581 cm^−1^) and A (593 cm^−1^) symmetries. From the studies by Tsuda and Arendts [49] and Kirchner et al. [50], it can be supposed that a maximum of 581 cm^−1^ oscillation income is due to the excitation occurring along “c” axis of apatite crystal. Since the irradiation is perpendicular to the sample in arrangement described in this contribution, “c” axes must be fixed parallelly to the DEJ-dentin boundary. It does not concern the interior of the DEJ and the DEJ-enamel boundary.

### 2.6. General Remarks

**Figure 9:**
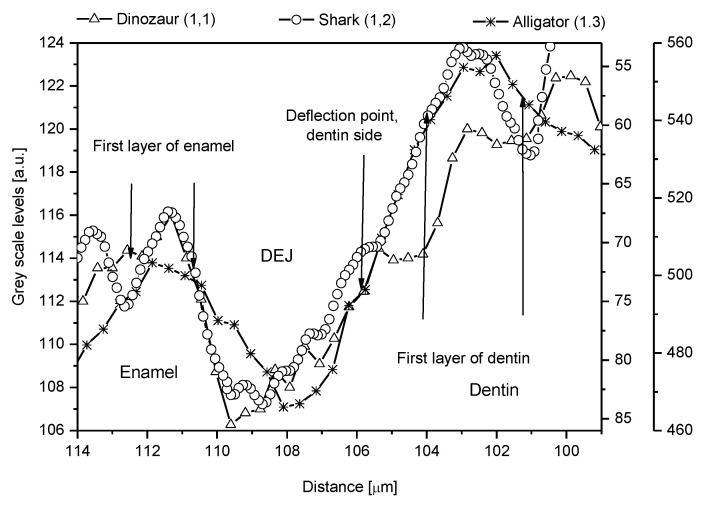
Outline of the DEJ zone for an unidentified American dinosaur, a shark, and an alligator.

We determined the values of parameters characterizing the DEJ using the PAP quantification for our EPMA, Johansson quantification for PIXE, and extracting density data from the transmission measurements. The information about the mechanical parameters and density is from Marshall [12] and Weatherell [35], respectively. The results are presented in Table 1. It is clear that the inorganic material of that zone is much closer to the mantle dentin, when it concerns the numerical values of parameters, than to the enamel. In that sense, it is even unusual that one can so clearly identify this zone in an optical image against the dentin. Perhaps the absence of water in this zone, as detected by Kolmas [38], plays some role in the process of visualization. It should be pointed out that the continuity of apatite matter is preserved in each point. Although the results of our measurements are specific to the samples, the general tendency is common in teeth. Figure 9 shows that this structure is more common in nature. Here, we made measurements on teeth of genetically different organisms as diverse as extinct dinosaurs and very old but still living species such as sharks and alligators.

The drop density of dentin relative to enamel is much higher than it results from simple comparison of the apatite/organic matter + water contents in dentin and enamel, respectively (~95%). It probably results from the loosened structure of collagen-apatite assemblies and the voids.

It seems that the strict determination of size, structure, and role of the DEJ can help in synthesis of the next generation of smart dental restorative materials, the internal structure of which would be tailored to the natural chemical and mechanical properties of each part of the tooth. A part of the recent failures should be ascribed to the inconsistency of fillings with the remaining tissue. This worsens the adherence of the new material to the original tissues. In addition, it gives catastrophic effects in the case of great mechanical stresses. Lastly, one should remember that the energetic status of apatite crystals in different locations is significantly different, as shown for the first time.

## 3. Materials and Methods

### 3.1. Materials

Samples of mature and sound molar teeth were collected. There were 12 samples from cosmetic treatments and accidents. All were collected from people in the age range of 20–30 years. The samples were cut with a diamond saw into thin (250 µm) but still stable slices just before the experiments. One side was polished with the diamond polishing wheel up to the moment when roughness was below 1 µm. The potential particle-shaped impurities in the preparation process were removed by ultrasound treatment (using ultrasonic cleaning baths with deionized water). The slices were shortly kept in slightly buffered water (0.1 M of Sorenson P5244 buffer) to avoid the sorption of any component of the potential buffer, both of cationic character and of potentially coordinative action (anions). The measurements were made within a few days after the preparation of the samples. Immediately before the measurements, the slices were taken from the water and carefully dried. The samples were mechanically stable, and the results were repeatable if the different kinds of measurements were performed in a relatively short time and in the correct order. We assured the repeatability of results by making observation of the same sample using the optical microscope in air, by SEM under vacuum, by Raman microscope in air, and finally by PIXE in vacuum (~10^−6^ Torr). The samples were placed in a movable and calibrated sample holder to repeat the measurements at the same or rather as close paths as it was possible. All the details observed in the first measurement by the optical microscope were still observed in the elemental results from the final analysis by PIXE. After measurements with PIXE, the samples were not used anymore due to the formation of small craters.

Several microprobes were used for studying the distribution of the inorganic and organic components in the DEJ region of the human molar teeth: EPMA, µ-PIXE, and Raman microscopy. The optical microscopy coupled with an image analysis program was applied for obtaining the topographical images of the samples.

### 3.2. EPMA

EPMA is a complementary device in relation to the scanning electron microscope (SEM). One can obtain both linear scans and elemental mappings for the selected characteristic X-Ray lines. To locate the scans or mappings in the relevant locations on the sample, one should collect the image of the sample in backscattered (or secondary) electrons and optionally add the optical image from the optical microscope. To extract the optical line scan from the image, the images were processed by MicroImage 4.0 program (Media Cybernetics, Rockville, MD, USA). Images, scans, and mappings had to be rescaled to the uniform length units. LEO 1430 VP SEM was applied in our research. The function of the electron microprobe was executed by the attachment of the Si(Li) detector (Röntec, Germany). The energy resolution was measured for MnKα line and was established as 180 eV. The settings for the measurements were fixed as 20 keV accelerating voltage and 0.7 nA beam current. The conditions of the experiment allowed measuring both very light elements, like carbon, and much heavier ones, like calcium. To remove the electric charge from the sample surface, the sample was supported with the conductive glue produced by the Agar Scientific company. The lateral spatial resolution of the measurement was determined as ~1 µm, and the steps in the scan operation were set as 1 µm. We applied Pouchou and Pichoir (PAP) procedure [51] for quantification of the chemical composition. Pure hydroxyapatite samples served as a single standard sample.

### 3.3. µ-PIXE

µ-PIXE experiments were carried out at the Henryk Niewodniczański Institute of Nuclear Physics, Polish Academy of Sciences, Cracow, Poland. The selection of this method was aimed at trace element analysis due to the much lower Bremsstrahlung-type background in PIXE with respect to EPMA. The total length of the microprobe facility, supported on the Van der Graaf accelerator, was 230 cm. Two doublets of magnetic quadruple lenses, called “Divided Russian Quadruplet”, were the basis for the proton focusing optics. The beam of protons with an energy of 2.5 MeV was deflected by ca. ±125 µm in two dimensions, enabling the scans and mappings. The spatial resolution of mappings was delimited by the beam spot on the target which was in the range of 3 µm × 3 µm, with the object aperture of 50 µm, and the angular collimator of 200 µm. The beam current centered on the target point was ~100 pA. A detailed description of the Cracow PIXE device was given in a paper by Lebed et al. [45]. The single-step size in the scan operation was set as 1.25 µm. X-Ray data were collected by the PGT Si(Li) detector (80 mm^2^, 162 eV at 5.9 keV) in a proprietary, multiparameter data acquisition system [52].

### 3.4. Micro-Raman

Micro-Raman experiments were made with a Renishaw inVia Reflex spectrometer, with a Leica (Wetzlar, Germany) stereoscopy microscope attached, with a ×50 objective. The spectrometer was equipped with a liquid nitrogen-cooled CCD detector. For the excitation, a laser diode emitting radiation in the IR region, with a wavelength of 785 nm, was applied. The applied power of the laser was 300 mW. The teeth samples were analyzed using a Renishaw SynchroScan mode from 400 to 3200 cm^−1^ with a spectral resolution of about 2 cm^−1^. The beam was focused on the ~1 µm wide spots on the sample, and the steps were taken as 1.61 µm. The observed lines were: ν_1_ symmetric stretching line for PO_4_^3−^ ion ~960 cm^−1^; ν_4_ mode for PO_4_^3−^ ion 587 cm^−1^; 1070 cm^−1^ for CO_3_^2−^ line, substitution B; 3372 cm^−1^ for NH stretching line. The initial treatment of data was made using the Wire2 computer program and completed with Origin 8.1 software. The areas of overlapping peaks were calculated with Lorentzian deconvolution functions.

### 3.5. Micro-XRD

The point phase analysis of the chemical structure of the teeth was carried with the use of a tabletop XRD device—Philips X’Pert MPD, with Cu tube, the photon output of which was collected and guided to the sample with the capillary of 50 µm-wide diameter. The sample was shifted with steps ~30 µm long. It slightly improved the spatial resolution of results which was between 30 and 50 µm and as we will see, to an insufficient degree (Figure 6a,c). The obtained data were identified with the use of JCPDF (International Centre for Diffraction Data Files). The spectra were collected within the angular range of 5–60 degrees of 2θ angles. The lower angular limit was set to detect the X-Ray scatterings from the organic matter. The crystallographic parameters “a” and “c” for the hexagonal structure were calculated according to normal procedures [53]. The diffraction results enabled our original, first in the world scale, calculation of energy necessary for apatite and crystallite transformations.

### 3.6. Optical Microscopy

All the samples of teeth were observed in parallel using the Eclipse E400 (Nikon Europe B.V., Amsterdam, The Netherlands) optical microscope, and the selected images were fixed using the attached Coolpix 950 digital camera (Nikon Europe B.V., Amsterdam, The Netherlands). The images were collected as files. The analysis of images was performed using the image-processing MicroImage 4.0 program. The images could be compared with the results of chemical mappings from the electron microprobe. When the function of the linear profile extraction was used for photos, the resulting “optical scan” could be easily compared with the chemical scans [49], and this option was preferred due to its demonstrativeness and easier conversion to a digital form. The lateral dimension of the pixel was equal to 0.78 µm.

### 3.7. External Data

Since we did not perform the micromechanical measurements, we contacted Prof. Sally Marshall from the University of California, School of Dentistry, San Francisco, USA, from whom we got the permissions to tailor her and her colleagues’ results to our microchemical data. Their data [44] were collected for the limited number of incisors and molars, for which they measured the elastic modulus and hardness using the standard AFM, with the head replaced with the capacitive sensor. They also measured the friction across the DEJ using the nanoscratch tester with two capacitive sensors for detecting the vertical and lateral displacements under the influence of the applied force.

During work with all spectra, we used the Origin 8.1 software for calculation of relative energy variations and estimation of correlations between variables.

## 4. Conclusions

A very clear image of the DEJ emerges from our microchemical and microoptical studies with the support of micromechanical results by Marshall et al. [12] and by comparison with the results by Fong et al. [11]. To our knowledge, it is the first clear and comprehensive delimitation in the world literature, even if some other comprehensive papers arrived [44]. The DEJ zone is precisely defined by the chemical, mechanical, and optical properties as well. The 4% drop in the Ca contents corresponds to a 2.2% drop in the P contents, and it translates to an 87.5% drop in hardness and a 30% drop in the elasticity modulus; Marshall et al. [12], Figure 7 therein. It occurs at the 15 µm distance. As it is shown in Figure 8, the correlation between the decrease in the Ca and P contents and the hardness drop is strong and clear, and one can make it coincide with breaking the rod-like shape of the hydroxyapatite crystals. When crystals are in a rod shape, they adhere to one another very compactly and are difficult to break. It suggests that the total reorganization in the shape of the hydroxyapatite crystals occurs within some 15 µm on the enamel-DEJ boundary, and it corresponds to the zone of elevated Mg and Na concentrations which prolongs into half of the DEJ. The presence of Mg and Na can be attributed to the crystallographic changes inside hydroxyapatites (see [54]).

The Mg and Na-enriched apatites fill the zone in the region adhering to the enamel-DEJ zone (Figure 9). They most probably participate in the reorganization of the crystalline material from the grain- to the rod-like crystallites [55,56,57,58]. 

Another significant achievement resulted from a totally novel method of energy calculation of crystallographic transformations inside apatites. It was proved that the main energetic change occurs along the dentin-DEJ boundary, and this released energy is a large amount. The DEJ position is at the same time the location of minimum energy for the transformation of apatites, and this fact evidences energetical stabilization of the zone.

There are very strong indications that the structure of the DEJ is universal, quite independent of the kind of tooth and in many cases of the kind of the animal ([7,8,9] and our Figure 9). One can carefully couple the data taken from different measurements for different samples and made with the use of different methods.

## Figures and Tables

**Table 1:** Basic values of parameters for enamel, DEJ zone, and dentin; data for density adapted from Weatherell [35] and for hardness, elasticity modulus, and friction from Marshall et al. [12].

	Density [g/cm^3^]	Hardness [GPa]	Elasticity Modulus	Friction	P [%]	Ca [%]	C [%]
Enamel	2.85	3.2	75		17.2	34.8	2 (19%)
DEJ	2.1 (73.7%)	0.4 (12.5%)	22 (29.3%)	2.1 (36.2%)	15.0 (87.2%)	30.8 (88.5%)	
Dentin	2.2 (77.2%)	0.6 (18.75%)	26 (34.7%)	5.8	13.6	31.2 (89.7%)	10.5

## Data Availability

The raw/processed data required to reproduce these findings cannot be shared at this time as the data also form part of an ongoing study.
